# “Messing with the mind”: evolutionary challenges to human brain augmentation

**DOI:** 10.3389/fnsys.2014.00152

**Published:** 2014-09-30

**Authors:** Arthur Saniotis, Maciej Henneberg, Jaliya Kumaratilake, James P. Grantham

**Affiliations:** ^1^Biological Anthropology and Comparative Anatomy Unit, School of Medical Sciences, The University of AdelaideAdelaide, SA, Australia; ^2^Centre for Evolutionary Medicine, University of ZürichZürich, Switzerland

**Keywords:** brain size, hominin brain, memory formation, brain-machine interfaces, nootropic agents

## Abstract

The issue of brain augmentation has received considerable scientific attention over the last two decades. A key factor to brain augmentation that has been widely overlooked are the complex evolutionary processes which have taken place in evolving the human brain to its current state of functioning. Like other bodily organs, the human brain has been subject to the forces of biological adaptation. The structure and function of the brain, is very complex and only now we are beginning to understand some of the basic concepts of cognition. Therefore, this article proposes that brain-machine interfacing and nootropics are not going to produce “augmented” brains because we do not understand enough about how evolutionary pressures have informed the neural networks which support human cognitive faculties.

## Introduction

The issue of brain augmentation has received considerable scientific attention over the last two decades. Much of this focus has been prompted by the increase in human aging population and the concomitant rise in dementiae and neuro-degenerative disease. Moreover, brain augmentation has become a central theme for transhumanists who argue for the creation of various bio-technologies in order to transcend the limitations of the biological body (Drexler, 1992; Roco and Bainbridge, 2002; Bostrom, 2003; Ramez, 2005). Kurzweil (2000) has suggested that development of brain-machine interfaces will be necessary to cope with the informational demands of future high tech societies. He even proposes the supplanting of a “cognitively superior” nanotech brain to supplement the biological human brain (Kurzweil, 2000). Transhumanists have overlooked the complex and plural selective pressures which have led to the human brain’s current functioning. Like other bodily organs, the human brain has been subject to the forces of biological adaptation (Hawks et al., 2007; Henneberg and Saniotis, 2009), thus it is continuously changing. The challenges that humans are faced with are the continuously changing living environment and to a very much lesser extent the “technological advancement”. Being a result of the trial-and-error processes of biological adaptation the structure and function of the human brain are very complex and only now we are beginning to understand some of the basic concepts of cognition (i.e., in relation to memory, memory consolidation and retrieval). Therefore, this article proposes that brain-machine interfacing is not going to produce “augmented” brains because we do not understand enough about how evolutionary pressures have informed the neural networks which support human cognitive faculties.

## Evolution of human brain size and intelligence

There is no doubt that humans display behavioral complexity greater than other mammals, and there is ample archeological evidence for the historical development of human mind that is a system of informational processes manifesting itself in symbolic communication transmissible from one individual to others (Bednarik, 1997; Butler, 2005; Bar-Yosef, 2007; Burke, 2010; Lycett and Chauhan, 2010). However, it is still difficult to pinpoint and identify what is special about a biological substrate that led to the evolution of human complex behaviors.

**Figure 1:**
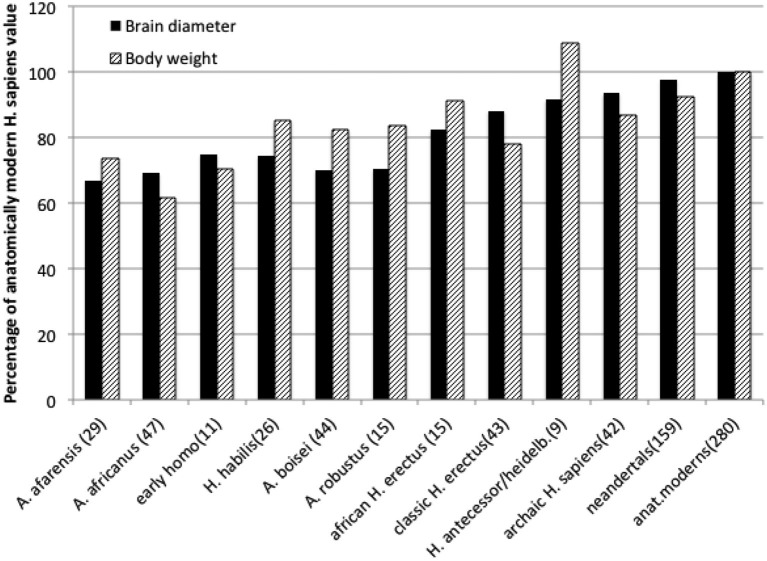
**Average brain diameters (a cubic root of endocranial capacity) and estimated body weights of hominids expressed as percentages of anatomically modern human averages**. Data from De Miguel and Henneberg (2001) and Mathers and Henneberg supplemented with newer finds. Numbers in brackets are numbers of individual estimates taken into account.

Human brain is a mammalian organ that in no single particular way is exceptional. Its anatomy is very similar to that of other primate brains (Radinsky, 1979). For the long time it has been widely accepted that during several million years of hominin evolution the human brain became especially large, thus indicating anatomical basis for our unusual abilities. This, however, turns out not to be true at closer scrutiny of the fossil record of hominin evolution. True enough, the volume of hominin braincase tripled in the last, approximately, 3 million years (from about 450 ml to current 1350 ml, De Miguel and Henneberg, 2001). During that time, however, hominin body size increased, too. Body size is measured either as the linear height, or weight, that in humans scales approximately to the second power of height (Henneberg et al., 1989), a fact generally recognized by the construction of the Body Mass index as a ratio of weight to height squared. When the size of human brain is expressed as a linear dimension (a cube root of volume), its increases over the last 3 million years are comparable to those of height (Henneberg and Saniotis, 2009) and weight (Figure 1). The size of the human brain is proportional to the size of musculoskeletal system mass (Rogers, 1992); scaling of human brain size to body size, in contrast to other vertebrates and mammals, where brain size increases allometrically at a fraction of body size (Jerison, 1973; Martin, 1990) is isometric, due to changes of body structure related to erect bipedalism, high quality diet and extraoral food processing together reducing body size (Henneberg, 1998). Physiological regulation of the human brain by endocrine exchanges follows the same principles as that of all other mammals, but the quantities of specific active substances may differ (Previc, 1999, 2009).

The size of the human brain does not correlate meaningfully with the mental abilities (Henneberg et al., 1985; McDaniel, 2005); the size explains at best about 10% of the variation in “intelligence” and even this number is debatable. Higher intellectual functions are difficult to localize precisely to specific regions of the brain compared to processing of sensory inputs and motor outputs (Power et al., 2011), while many tasks controlled by brains are processed in complex networks widely distributed across the cortex (Bullmore and Sporns, 2009). Researchers have tried to justify human uniqueness, since the rise of modern scientism by quoting various “exceptional” human brain characteristics. These were mostly associated with anatomical character due to the slow progress in physiological research on the human brain (because of ethical constraints), especially those related to neurotransmitter and hormone regulation of the central nervous system functions (i.e., primarily, synaptic function). The most common among the indices defining the uniqueness of the human brain are variously constructed “encephalization” indices. They combine in various forms information on brain size and body size, with the assumption that mammalian brains need to have a certain number of neurons to receive sensory information and process it to control functions of the body. The prevailing hypothesis states that the larger the brain in relation to body size, the greater the ability to process information. Averages of encephalization indices calculated in various ways (Table 1) place humans clearly above other mammals and human ancestors. However, when the range of variability in human brain size and the body size is taken into account differences between individual humans may be greater than those between Australopithecines and modern *Homo sapiens*. South African *Australopithecus robustus* had 4.3 × 10^9^ extraneurons, while modern *Homo sapiens* have 8.2 × 10^9^ extraneurons (McHenry, 1976, extra-neuron numbers were calculated using Jerison, 1973 formula). The large difference of 3.9 × 10^9^, however, is smaller than the differences between some modern humans (see range in Table 1). When normal intraspecies variation is taken into account, it follows that individual members of our species, *H. sapiens*, do not differ from some individuals of *H. erectus* since the ranges of encephalization indices of these two species overlap widely.

**Table 1:** **Ranges of various indices of encephalization in modern humans expressed as the lower and upper limits of 99% confidence intervals and their midpoints compared with *Homo erectus* midpoints (data from Henneberg, 1990) supplemented by *H. erectus* ranges calculated in a similar way using data from Henneberg and Thackeray, 1995**.

Index (Author)	*H. erectus* lower limit	*H. erectus* midpoint	*H. erectus* upper limit	*H. sapiens* lower limit	*H. sapiens* midpoint	*H. sapiens* upper limit
Extraneurons (Jerison, 1973)	5.96 × 10^9^	7.02 × 10^9^	8.38 × 10^9^	6.94 × 10^9^	9.17 × 10^9^	11.39 × 10^9^
Encephalization Quotient (Jerison, 1973)	3.70	5.51	8.11	5.64	8.51	11.38
Index of Progression (Stephan, 1972)	18.59	23.0	32.33	23.6	35.0	46.4

*Note that H. erctus midpoints are close to H. sapiens lower limits while H. sapiens midpoints are close to H. erectus upper limits. There is an obvious overlap between ranges of the two taxa*.

Brains of different species have different neuronal densities and different levels of myelination in various regions (Haug, 1987; Glasser et al., 2014), which means that bigger brain does not necessarily contain more neurons. Usually neuronal density decreases with increasing brain size (Haug, 1987).

## Brain-machine interfaces: evolutionary challenges

Animal research in brain-machine interfaces has led to an improved understanding of memory and sensory processes and neural firing patterns, leading to possible prosthetic therapies for the restoration of motor function (Nicolelis, 2003; Nicolelis and Srinivasan, 2003; Sanchez et al., 2003; Lebedev and Nicolelis, 2006; Moritz et al., 2008; Ethier et al., 2012). These developments have prompted some thinkers to suggest that humans are on the verge of a “technological cognitive revolution” (Nicolelis and Srinivasan, 2003). Current research is focusing on the use of computers to ascertain information on a user’s cognitive state by observing their physiology (Tan and Nijholt, 2010), thus therapeutically directed brain-machine interfaces appear to be promising (Collinger et al., 2012; Shih et al., 2012; Borton et al., 2013; Ifft et al., 2013; Thakor, 2013; Raspopovic et al., 2014). Recent developments have focussed on stimulation of ulner and median nerve fascicles using transversal multichannel intrafascicular electrodes, which enables an amputee to adapt their grasping force (Raspopovic et al., 2014). In another recent study a bimanual BMI has been developed that enables rhesus monkeys to simultaneously control two avatar arms. The bimanual BMI is based on extracellular activity of 374 to 497 neurons monitored from various parietal and cortical areas (Ifft et al., 2013). These developments should assist in the design of BMIs which enable human patients better manual control.

While neuroscience research is advancing BMI therapeutic capabilities, there is yet no existing brain-machine interface based on exchange of electrical (electromagnetic) signals that would improve human cognitive abilities above and beyond what a natural brain can do. We do not have yet a theory correctly approximating physical substrate of higher cognitive processes. Brain did not evolve by adding defined units for more complex functions, it improved its performance by physiological modulation enabled by biochemical alterations of neuroactive substances.

Therefore, the belief that brain-machine interfaces offer a viable method for augmenting cognitive processes lacks scientific credibility (Kurzweil, 2000). The mainstay of this rhetoric has come via futurists who have generally ignored evolutionary processes, which have produced the current structure and functions of the human brain. What we have to remember is that our advanced technology that is in use in society is not the product of the brain of one person, who generated it in a short time. Rather it is the combined effect of multitudinous brains over a long historical period (i.e., learning, processing of learned information, researching and planning) together or separately over a long period of time. The brain is a unique organ that “changes” with learning and processing of the learned material to generate novel ideas that could be researched or tested. In short, the brain is continuously changing to generate the complex technological advances of the modern world. Therefore, a normal brain does not need a brain-machine interface to cope with the ever increasing technology or new information; what the brain needs is continuous input of the new information (i.e., learning). Furthermore, when an individual executes an action (mental or physical), it results from the complex interactions of information inputs and outputs from many regions of the brain (e.g., a muscular action—sensory cortex, motor cortex, basal nuclei, cerebellum, etc.) and the level and type of interaction from each region vary (Blumenfeld, 2010; Michael-Titus et al., 2010). Therefore, proper understanding of all these process is critical, before manufacturing “brain-machine” interfaces to augment brain functions. If this path is not taken, brain-machine interfaces could cause more harm than benefits.

We may infer from this that any attempts to augment human intelligence via brain-machine interfaces will be problematic due to evolutionary dynamics underpinning the human brain. Furthermore, the incredibly complex nature of neural networks, chemical complexity of nerve signals conduction and individual anatomical and physiological variation pose enormous challenges for interaction of engineered devices with association networks in the human brain. However, in the current environment, brain-machine interfaces may have some therapeutic benefits in individuals developing dementiae, neuro-degenerative diseases and sensory input inadequacies (blindness, deafness). For example, neuromodulation using deep brain stimulation (DBS) is currently being used to reduce Parkinsonian symptoms in selected patients. A goal of DBS is not merely to slow down cognitive decline, but also to lead to a restoration of function, thereby increasing life quality (Zibly et al., 2014). Advantages of DBS surgery are its low complication rates and comparatively higher safety levels when performed by expert neurosurgeons (Zibly et al., 2014).

Since complex cognitive tasks rely on widely dispersed intersecting neural networks involving various parts of the brain, it is thought to be difficult to connect to the brain an engineered device that would assist or augment complex thoughts. Since transmission of signal from one neuron to other neurons is mediated chemically, it may be more feasible to introduce into brains substances that alter the efficiency of neurotransmission. Chemical engineering may be more efficient than electronic engineering.

## Nootropic agents and evolution

There are a number of chemically based methods of augmenting the human brain, forming an important element of cosmetic neurology (Dees, 2004). The field of cosmetic neurology is increasingly dependent on the development and application of nootropic agents (Cakic, 2009). A nootropic agent is a substance that may alter, nourish or augment cognitive performance, predominantly through the stimulation or inhibition of certain neurotransmitters (Nishizaki et al., 1999). These agents may occur in nature or be synthetically derived (Dielenberg, 2013). These substances have been proven to increase concentration, harness memory potential and expedite cognitive functioning (Turner et al., 2003, 2004). Many of these agents act under the premise of manipulating neurochemistry in a targeted fashion and are predominantly stimulatory in nature (Copani et al., 1992). Most traditional and modern nootropics activate an excitatory neurotransmitter or suppress the action of its inhibitory counterpart (Ito et al., 1990; Nicoletti et al., 1991; Staubli et al., 1994; Lynch and Gall, 2006; Huff, 2012).

Many authors support the co-evolution of early hominins with the use of nootropic substances and the attainment of altered states of consciousness (Winkelman, 2000, 2001; Sullivan and Hagen, 2002; Saniotis, 2010). Indeed, the desire to augment cognitive performance through the consumption of particular substances predates antiquity.

There is evidence to support the long-standing and widespread use of nootropic agents on every inhabited continent. For example Aboriginal Australians have used the stimulatory effects of *Nicotiani gossei* for millennia (Watson, 1983; Sullivan and Hagen, 2002). The use of tobacco throughout North and Central America has been well established and coca, the pre-cursor of cocaine, was cultivated along the western coast of South America as long as 7,000 years ago (Balick and Cox, 1996; Sullivan and Hagen, 2002). This cultivation ran contemporaneously with the use of cannabis in Europe (Schultes and Hofmann, 1979). The modest potency of organically derived substances and the long-standing, stable use of the aforementioned products in these ancient cultures proved to be beneficial (Saniotis and Henneberg, 2011). The strongest evidence to support this development is the presence of encoding DNA specific for the metabolization of these substances, such as the cytochrome P450 2D6 (CYP2D6) gene (Saniotis and Henneberg, 2011). These examples illustrate the inextricable involvement of environmental substances in altering or augmenting cognitive performance.

The affiliation with biological neuro-stimulants has continued through to recent history. The industrial revolution permitted the production of mind-altering substances on an unprecedented scale. Throughout the 20th century, there was a proliferation of synthetically derived substances applicable to cosmetic neurology. Today, nootropic agents are used to intentionally augment cognitive performance. University students appear to be amongst major perpetrators as they complete assignments and prepare for examinations (Greely et al., 2008). Prescription medications such as methylphenidate (Ritalin) and dextroamphetamine (Adderall) are being increasingly used and modafinil, an analeptic prescription medication has been used as a study aid by one-fifth of UK university students (Ghahremani et al., 2011; Fitzsimons and McDonald, 2014). Modafinil is believed to increase concentrations of glutamate and decrease GABA within the posterior hypothalamus, producing an overall neuro-excitatory effect (Ferraro et al., 1999). The drug has been shown to improve attention and working memory in medical practitioners and aviators and may be used in other challenging professions (Turner et al., 2003; Chatterjee, 2004; Müller et al., 2004; Walsh et al., 2004; Czeisler et al., 2005; Warren et al., 2009; Garcia et al., 2013).

However, the over-application of modern cosmetic neurology is fraught with danger and has been proven deleterious in many instances. In the short term, modafinil is known to produce nausea, vomiting, diarrhea, dyspepsia, headache, insomnia and anxiety with its long-term complications remaining largely unknown (Ballon and Feifel, 2006; Sahakian and Morein-Zamir, 2007). More alarmingly, the consumption of commonly used psycho-stimulants, including Ritalin and Adderall, has been linked to the precipitation or exacerbation of underlying mental illness, sleep disturbances and cerebrovascular disease (Cakic, 2009). This is likely due to the modern human brain being maladaptive to the exaggerated pharmacological alteration of neurochemistry (Sullivan and Hagen, 2002). Increased drug potency associated with synthetic production has outpaced the brain’s capacity to metabolize and clear toxic substances, leading to prolonged exposure to these potentially harmful products (Sullivan and Hagen, 2002). This may be an example of evolutionary mismatch (Sullivan and Hagen, 2002). Whatever the mechanism, it appears these ill-adapted responses to modern nootropic agents may account for the bulk of the observed negative outcomes.

The potential scope of application for nootropic agents is vast. Mind-altering substances have the capacity to optimize cognitive performance and maximize human achievement. However, the limits of pharmacologically aided human cognition should not exceed the capacities of the brain. The human brain is a complex organ, thus pushing its performance beyond its adaptive capacity using pharmacological products could lead to failure. Therefore, caution must be taken when approaching the inherent risks of exacerbating the existing evolutionary mismatch in order to avoid deleterious outcomes. The majority of these outcomes are likely to relate to unbalancing salubrious and delicate neurochemical concentrations. Many psychiatric conditions, including schizophrenia, bipolar disorder and major depression, have illustrated neurochemical etiologies (Knable and Weinberger, 1997; Hirschfeld, 2000; López-Figueroa et al., 2004; Berk et al., 2007). Evolutionary challenges aside, there are also unresolved ethical and practical issues related to the intentional consumption of nootropic agents, not the least of which being whether it is fair, ethical and sensible to do so.

## Conclusion

Many believe it to be evident that the human brain has a tremendous propensity for technologically driven augmentation. Several authors have discussed the potential for anatomical and physiological enhancement via brain-machine interfaces and cosmetic neurology. Despite the hypothetical applications of cognitive improvement, this article has argued that human brain augmentation possesses a number of inherent challenges, many of which are informed during prehistory. The daunting complexity of neurological processes which inform cognitive abilities, combined with a current lack of understanding will likely confound any attempts in creating “smarter” minds. In fact, any attempt to circumvent the archaic substructures of the human brain may only serve to exacerbate the already existing maladaptive responses. For this reason, great caution must be adopted in approaching further attempts to go “messing with the mind”.

## Conflict of interest statement

The authors declare that the research was conducted in the absence of any commercial or financial relationships that could be construed as a potential conflict of interest.
